# Facilitators and barriers of Community Case management of Malaria implementation in Homabay, Busia and Kakamega Counties, Kenya

**DOI:** 10.1371/journal.pone.0329709

**Published:** 2025-08-21

**Authors:** Sheila Lumumba, Albino Luciani, Bryson Sifuma, Dennis Kinyua, George Gikunda, Norah Ogutu, John Onyango, Diana Mukami, Colleta Kiilu, Saida Kassim, Yvonne Opanga, George Githuka

**Affiliations:** 1 Amref Health Africa, Institute of Capacity Development, Nairobi, Kenya; 2 Kenya Medical Research Institute, Center for Global Health Research, Nairobi, Kenya; 3 Amref Health Africa in Kenya, Nairobi, Kenya; 4 Ministry of Health, Division of National Malaria Programme, Nairobi, Kenya; Sunu Sante Consulting, SENEGAL

## Abstract

**Background:**

Community Case management of malaria (CCMm) is a strategy used in malaria-endemic areas to reduce malaria morbidity and mortality. CCMm involves providing malaria diagnosis and treatment within the community by trained community health volunteers (CHVs). While evidence suggests CCMm is effective in combating the disease burden at the community level, it isn’t without challenges. This study assesses facilitators of and barriers to uptake of CCMm.

**Methods:**

This cross-sectional study employed a mixed methods approach. Quantitative data was collected using a household questionnaire targeting 528 participants, while qualitative data was collected using 4 focused group discussions and 20 key informant interviews. Quantitative data was cleaned, coded, and analyzed using STATA version 14. Qualitative data was transcribed, and the data was analyzed using NVIVO version 10.

**Results:**

The study found that 72% of households had received a service on Malaria, and this was consistent across all counties (Busia 75%, Homabay 72%, Kakamega 71%). 62% of respondents considered CHVs a regular source of healthcare, with approximately 85% of the population being satisfied with the services offered by CHVs. Key initiatives that improved the effectiveness of CCMm included sensitization on malaria causes and preventive measures, training of CHVs on the management of malaria, and empowerment of CHVs who can utilize rapid tests to diagnose malaria at the household level.

The facilitators of CCMm included the availability of malaria commodities, a functional referral system, and support supervision from the Community Health Assistants (CHA) and Health Management Teams. Barriers that hindered the implementation of CCMm included myths and misconceptions surrounding the use of mosquito nets, stock outs of malaria commodities such as the Malaria Rapid Diagnostic Test (mRDT) kits and antimalarials, and inaccessible roads into the communities.

**Conclusion:**

In spite of great strides in CCMm initiatives to reduce malaria-related Morbidity and mortality, some of the barriers underpinning its effectiveness remain unaddressed. Continuous training for CHVs, sustained availability of commodities for testing and treating malaria, and incentives are essential for the success and sustainability of CCMm initiatives.

## Background

Malaria remains a major public health and socio-economic problem in Kenya, accounting for 13% to 15% of outpatient consultations [1]. Despite significant strides in reducing malaria cases, 70% of the population is still at risk. The prevalence of malaria varies by region, with the highest prevalence observed in the Lake endemic region, 22.4% [1]. Kakamega, Homabay, and Busia Counties are among the counties with the highest prevalence of malaria in Kenya. A study in 2019 found that the prevalence of malaria in Kakamega County was 33%, Homabay 21%, and Busia 37% [2].

In 2018, the Division of National Malaria Program (DNMP) conducted a Malaria Program Review (MPR) that underscored the fact that coverage for malaria preventive interventions remained sub-optimal. Despite massive distribution, the findings indicated low coverage (76%) of long-lasting insecticidal nets (LLINs) against the National target of 80% [3]. Although mass distribution campaigns have increased the availability of Insecticide treated Nets (ITNs), usage remains low. The performance for the intermittent preventive treatment in pregnancy (IPTp) was below the national targets, with 70% of women receiving one dose, 56% receiving two doses, and 38% receiving the recommended three doses against the target of 80%. The MPR noted that sub-counties bordering lake endemic counties were not implementing IPTp and generally, there was late first presentation to antenatal care, leading to suboptimal IPTp coverage in the focus counties. On malaria diagnostics and case management, the MPR noted a significant improvement in the testing rate of suspected malaria cases in public health facilities, rising from 24% in 2010 to 64% in 2017. Among those diagnosed, 89% of confirmed malaria cases receiving appropriate treatment with artemisinin-based combination therapies (ACTs). The key issues identified included sub-optimal adherence to national treatment guidelines among healthcare workers in the public and private sectors and inadequate implementation of community case management for Malaria due to regulatory bottlenecks.

Community Case Management of malaria (CCMm) is one of the strategies adopted by malaria-endemic countries to combat the disease at the community level and reduce the burden on vulnerable populations [4]. CCMm is based on evidence that well-trained and supervised Community Health Volunteers (CHVs) can promptly and adequately treat fever cases within 24 hours to help reduce malaria morbidity and mortality among under-five children in Africa [5]. It enables timely diagnosis and treatment of uncomplicated cases of malaria by trained CHVs using malaria rapid diagnostic tests (mRDTs) and artemisin-based combination therapies (ACTs).

CHVs in Kenya, are individuals selected by their communities to provide basic health services at the household level. Working under the Ministry of Health, they are attached to community health units and supervised by Community Health Extension Workers (CHEWs). Their roles include health education, malaria testing, treatment of uncomplicated malaria, referral of severe cases and maternal and child health promotion.

The Kenyan government formally embraced CCMm with the roll-out of the Community Health Strategy (CHS) in 2006. This institutionalized the role of CHVs within the national health system. Pilot CCMm programs were introduced around 2010 and subsequently scaled up following the 2014 revision of the Community Health Policy. These efforts have been led by The Division of National Malaria Control Programme (NMCP) which oversees CHV training and supervision. Notably, previous studies have established that CHVs are up to the task of diagnosing malaria in rural and underserved areas, thereby contributing to health equity [6,7]. Despite significant progress, challenges such as commodity stock-outs, irregular CHV remuneration and uneven policy enforcement across counties have limited full national implementation and sustainable adoption of the strategy [8].

To help bridge these gaps, Amref Health Africa has been a longstanding partner in strengthening community-based health systems in Kenya. Over the past decade, Amref has played a critical role in scaling up CCMm across multiple malaria-endemic counties. Through collaborative efforts with MOH and county governments, Amref has supported the training, mentorship, and deployment of CHVs, improved access to malaria commodities and facilitated health education at the grassroots level.

One such initiative is a GSK-funded project that ran from January 2021 to August 2022. The project sought to strengthen the capacity and capability of the health system in the Western region of Kenya, thus catalyzing faster progress towards achieving the results articulated in the national Malaria strategies. To achieve this, the project focused on strengthening the community health strategy and provided training and mentorship to CHVs and CHEWs on CCMm to enhance community-based malaria interventions. Additionally, to boost the uptake of malaria interventions, the project supported the development and implementation of malaria advocacy, communication, and social mobilization to improve social and behavior change communication and enhance community awareness of the demand for and utilization of malaria prevention and treatment services. Social mobilization helps communities bring together allies to raise awareness of and demand for the Malaria programme, to assist in the delivery of resources and services, and to strengthen community participation for sustainability and self-reliance

Therefore, this study sought to establish the facilitators and barriers influencing the effectiveness of CCMm in Kakamega, Homabay, and Busia counties in Kenya.

## Methodology

**Fig 1:**
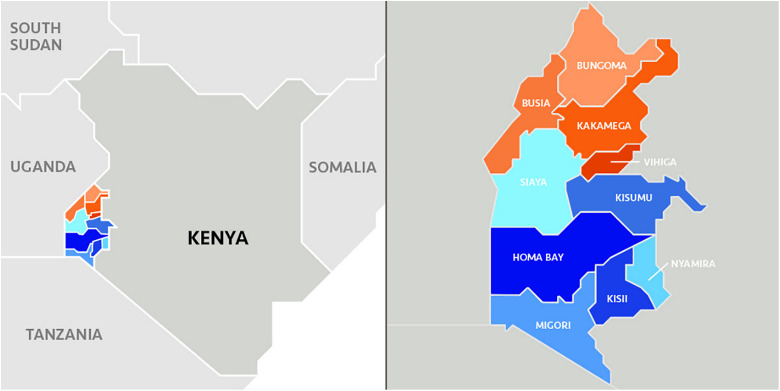
Map of Western Kenya.

The study adopted a cross-sectional study design using mixed methods of data collection. Quantitative data was collected through structured household surveys, while qualitative data was collected using Focus Group Discussions (FGDs) and Key Informant interviews (KIIs). The study was conducted in three counties located in the lake endemic areas of western Kenya, Kakamega, Busia, and Homabay (Fig 1). These counties were purposively selected due to their high malaria burden and representativeness to get a holistic view of the effectiveness of CCMm and barriers to the uptake of the services in Western Kenya.

The Cochran formula for single proportion allocation was used to determine a representative sample size for the household survey.


$$n=\;\;\frac{\left(Z^{\text{2}}\text{\textbackslash{} }p*\;(\text{1}-p\right)}{e^{\text{2}}}$$

Where,

n = sample size

Z = Z-score for a 95% confidence level: (1.96 standard value)

p = estimated proportion of households with suspected malaria: (assumed at 0.5 for maximum variability.)

e = margin of error: (0.05 standard value)

n= (〖1.96〗^2.0.5.(1-0.05))/〖0.05〗^2 =384


$$n=\;\;\frac{({\text{1}.96}^{2}\;.0.5\;.\;(1-0.5)\text{\textbackslash{} \textbackslash{} \textbackslash{} }}{0.05^{2}}=384$$

After adjusting for a 10% non-response rate, the sample increased to 422. To enhance representativeness across counties and account for design effects from sampling, the final sample size was set at 528 households. A multistage sampling approach was adopted. First the total population was stratified by county (Kakamega, Busia, Homabay). Then, the overall sample size of 528 households interviewed from 6^th^ April 2022–10^th^ April 2022 was proportionally allocated across the counties (n = 256 Kakamega, n = 155 Homabay, n = 117 Busia). Systematic samples selected from each stratum. The systematic sampling was based on households with individuals who had symptoms of Malaria.

The households were selected based on proportion of households with suspected malaria cases that had been referred to the health facility by community health workers and the population in the 3 counties. In each household only one respondent was interviewed. The eligible respondent was the primary caregiver of a child under five years of age. In this context, “caregiver” refers to the mother or legal guardian who is most responsible for caring for child’s daily care. If more than one eligible caregiver was present, the one most involved in the child’s healthcare was purposively selected by the interviewer.

The county sample size was further proportionally allocated to the sub-counties according to the 2019 Population and Housing Census, as in Table 1. Households were selected at every fifth interval if they met the inclusion criteria.

**Table 1:** Household sampling frame.

County	Sub-county	Total HHs	Sampled HHs
**Kakamega**	Matungu	36457	69
	Navakholo	32315	61
	Lurambi	39589	75
	Mumias West	26766	52
	**Total**	**135127**	**256**
**Busia**	Matayos	33160	38
	Bunyala	19039	22
	Butula	32213	36
	Bumala	19039	22
	**Total**	**103451**	**117**
**Homabay**	Rachuonyo East	27319	31
	Rachuonyo South	30990	35
	Suba South	27769	31
	Ndhiwa	48062	57
	**Total**	**134140**	**155**

For the qualitative component, purposive sampling was used to select participants based on their involvement in community health or malaria control. A total of 20 KIIs were conducted with County and Sub-county Health Management teams and 4 FGDs were conducted with Community Health Workers, as illustrated in Table 2 below.

**Table 2:** KII and FGD sampling frame.

Level	Respondent	Number
**County Health Management team (KIIs)**	County Malaria Coordinator	2
	County Director of Health	2
	County Disease Surveillance Coordinator	2
	County Health Promotion Officer	1
	County Reproductive Health Coordinator	1
**Sub-county Health Management team (KIIs)**	Sub-County Malaria Coordinator	5
	Sub-County Director of Health	1
	Sub-County Disease Surveillance Coordinator	3
	Sub-County Health Promotion Officer	1
	Sub-County Health Records & Information Officer	2
**Community (FGDs)**	Community Health volunteers (CHVs	3
	Community Health Extension Workers (CHEWs)	1

### Variables collected

The study collected the following variables as illustrated in Table 3 below. The complete anonymized dataset is available in S1 File.

**Table 3:** Summary of variables measured.

Variable Category	Specific Variables
Sociodemographic categories	Caregiver age, gender, education level, household size
Malaria -related behavior	Time to seek care after symptom onset, provider visited, adherence to referral
Community health interactions	Frequency of CHV visits, receipt of malaria testing, treatment by CHVs
Service uptake and access	Use of mRDTs, receipt of ACT treatment, completion of referral to health facility
Perception and awareness	Knowledge of malaria symptoms, trust in CHVs, satisfaction with CCMm services

### Data management procedures

Quantitative data was cleaned, and a master data sheet was created. Analysis was done using STATA version 14. Descriptive statistics were used to summarize and identify patterns in the data and develop key statistics and tabulations that indicate differences in different subgroups.

The KII and FGD audios were transcribed, and the transcripts were generated and cleaned. The qualitative data were analyzed using NVivo version 10 for thematic analysis. Codes were developed aligned with the study objectives. Emerging themes were used to triangulate the findings along the quantitative data and presented as narratives. The questionnaires and guides used in the household survey, FGDs and KIIs are provided in S2 File.

All data collected during the study is Amref Health Africa’s intellectual property. Therefore, it will be stored and archived according to Amref’s data management policy.

### Ethical considerations and procedures undertaken

Ethical clearance was obtained from the Amref Ethics and Scientific Review Commission (ESRC P1126/2022) and the National Commission for Science, Technology and Innovation (NACOSTI/P/22/16241). Permission was secured from the respective County and Sub-County health management teams.

Verbal Informed consent was obtained from each study participant. The consent process was guided by a standard informed consent script approved by the Amref Ethics and Scientific Review Committee (ESRC), under Approval Reference P1126/2022. Trained research assistants followed the script to explain the study’s objectives, potential benefits and risks and emphasized the voluntary nature of the participation including the right to withdraw at any time without any consequence.

To uphold ethical standards and ensure transparency, the consent process was witnessed by an unbiased adult nominated by the respondent, most commonly the household head. In instances where the household head was the respondent, they nominated another adult household member (aged 18 or older) to serve as the witness. The research assistant verbally explained the study details, and the participant provided oral agreement. The witness observed and confirmed that the participant’s consent was voluntary and that they understand what they are consenting to. Following the consent process, the witness excused themselves to ensure the interview was conducted in privacy and confidentially.

This process was documented through an audio recording of the consent discussion, along with written notes by the research assistant affirming that verbal consent had been obtained. All interviews were conducted in private settings. Research assistants signed non-confidentiality agreements prior to data collection, and all data collected were anonymized with no personal identifiers reported.

## Results

### Demographic profile of the respondents

A total of 578 respondents were interviewed. Most respondents (78%) were female compared to males (22%). Most of the respondents had primary level education (41%), 8% and 3% had college and university education, respectively, while 8% had never gone to school. Most of the respondents (80%) were married, as shown in Table 4 below;

**Table 4:** Demographic profile of respondents.

		Overall (n = 614)	Busia (n = 116)	Homabay (n = 147)	Kakamega (n = 315)
**Sex**	**Male**	22%	15%	26%	23%
	**Female**	78%	85%	74%	77%
**Respondent level of education**	**Never gone to school**	7%	8%	3%	8%
	**Primary**	48%	53%	60%	41%
	**Secondary**	35%	29%	31%	38%
	**College**	8%	8%	5%	9%
	**University**	3%	3%	1%	5%
**Respondent marital status**	**Married**	78%	75%	75%	80%
	**Single**	10%	8%	6%	12%
	**Divorced/ separated**	3%	8%	0%	2%
	**Widowed/ Widower**	10%	9%	19%	6%

### Effectiveness of existing malaria management initiatives

#### Improved knowledge, attitude, Beliefs and Practices.

All respondents (100%) were aware of Malaria. (97%) of the respondents indicated that Mosquito bites caused malaria. The other cause mentioned by 17% of respondents was cold weather, which was cited more in Busia (26%) than in Homabay (20%) and Kakamega (12%). A summary of the responses is included in Table 5.

**Table 5:** Causes of malaria.

Causes of Malaria	Overall (n = 614)	Busia (n = 116)	Homabay (n = 147)	Kakamega (n = 315)
Mosquito bites	97%	94%	98%	98%
Staying in the cold/ weather	17%	26%	20%	12%
Drinking dirty water	8%	18%	1%	7%
Being rained on	7%	6%	4%	9%
Dirty/messy/bushy environment	3%	11%	1%	1%
Stagnant/dirty water	3%	7%	2%	2%
Type of diet/food	1%	3%	1%	0%
Other	1%	3%	0%	0%
Don’t know	0%	1%	0%	1%

Most common symptoms of Malaria reported were; fever (89%), headache (71%), joint pains (61%), and vomiting (52%). Awareness of common symptoms is highest in Kakamega and seems to be lowest in Busia county. On beliefs on whether a healthy-looking person could have Malaria, majority (85%) of respondents agreed that a healthy person could have malaria.

#### Improved knowledge on methods of malaria prevention.

The survey sought to establish respondents’ awareness of the methods used to prevent Malaria, and the findings are captured in Table 6 below. The most common methods cited were sleeping under a mosquito net (95%) and draining of stagnant water (43%) with no major variations across the counties.

**Table 6:** Methods of preventing malaria.

Methods of Preventing Malaria	Overall	Busia	Homabay	Kakamega
Sleeping under a mosquito net	95%	95%	97%	94%
Draining stagnant water around the compound	43%	42%	39%	45%
Using mosquito repellents	16%	15%	21%	15%
Clearing bushes	6%	14%	3%	4%
Keeping warm	4%	10%	1%	4%
Spraying house	3%	0%	12%	0%
Self-cleanliness	3%	13%	1%	1%
Use antimalaria	2%	2%	2%	2%
Drinking clean water	1%	2%	0%	1%
Other	1%	4%	0%	0%
Don’t know	2%	2%	1%	2%

### Malaria services

Table 7 describes malaria services that CHVs offered in Busia, Homabay, and Kakamega. The most common service mentioned by all respondents was net distribution (71%). Followed by treatment using ACT or AL (45%), referral to a health facility (39%), testing (38%) and health education (19%). Comparison by County indicates that net distribution services were more common in Kakamega (85%) than in Homabay (69%) and Busia (77%). Notably, in Kakamega, the proportion of respondents that mentioned referral (40%) exceeded that of Homabay (37%) and Busia (37%). In contrast, treatment using ACT/AL as a service offered by CHVs was more common in Busia (77%) than in Homabay (57%) and Kakamega (32%), moreover, in Busia, more respondents (85%) mentioned having received testing service than those in Homabay (46%) and Kakamega (20%). In Homabay, more respondents received education services (38%) than in Busia (15%) and Kakamega (15%).

**Table 7:** Malaria services received by respondents.

	Overall	Busia	Homabay	Kakamega
**Net distribution**	71%	30%	69%	85%
**Treatment using ACT or AL**	45%	77%	57%	32%
**Referral**	39%	37%	37%	40%
**Testing**	38%	85%	46%	20%
**Health education**	19%	15%	38%	15%

The KII and FGD respondents noted several preventive measures available at the community level to prevent malaria. These include reported malaria cases in the household during the last month; nearly 7 in every 10 respondents (69%) had witnessed a malaria case in their households, and this was more prevalent in Busia (83%) than in Homabay (99%) and Kakamega (61%). Almost all (94%) participants had sought advice on malaria from a government hospital (52%). However, a comparison across the three counties indicated that more participants from Homabay (68%) and Kakamega (52%) compared to Busia (34%) sought help from government facilities. Notably, CHV advice was sought more in Busia (41%) than in Homabay (14%) and Kakamega (6%).

After falling ill, it was evident that support was sought from CHVs in Busia (39%) than in Homabay (13%) and Kakamega (6%). In comparison, visit to government hospitals were more common in Homabay (57%) and Kakamega (50%) than in Busia (31%) as indicated in Table 8.

**Table 8:** First point of treatment-seeking.

	Overall	Busia	Homabay	Kakamega
Government hospital/Health	47%	31%	57%	50%
Private hospital/clinic	15%	14%	12%	18%
CHV	15%	39%	13%	6%
Center/Dispensary	12%	8%	6%	17%
Private pharmacy	8%	8%	5%	9%
Shop	2%	0%	6%	0%

### Service provision by CHVs

**Fig 2:**
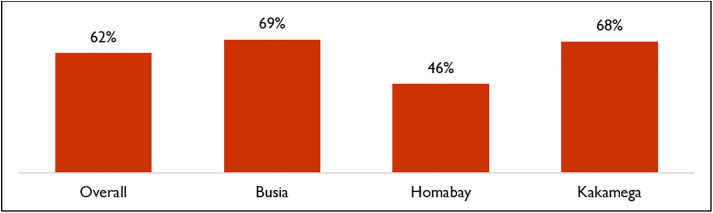
Respondents who consider a CHV as their regular source of health care.

Based on survey findings as shown in Fig 2, 62% of respondents considered CHVs a regular source of healthcare.

Table 9 below captures responses regarding the services that CHVs provided and is based on those who had received a service from CHV (n = 471). Majority (72%) had received services on Malaria, and this was consistent across all counties (Busia 75%, Homabay 72%, Kakamega 71%). Health education service (42%) and WASH services (41%) were more common. In contrast, Busia led other counties on CHV services regarding Maternal, Newborn, and Child Health (MNCH) (Busia 25%, Kakamega 11%, Homabay 8%).

**Table 9:** Types of services received from a CHV by respondents.

	Overall	Busia	Homabay	Kakamega
Malaria	72%	75%	72%	71%
Health education	42%	33%	60%	39%
WASH	41%	15%	34%	52%
Nutrition	26%	3%	15%	36%
TB	13%	4%	5%	17%
MNCH	13%	25%	8%	11%
HIV/AIDS	11%	0%	10%	15%
Provision of medicine/Vaccination (Malaria, Covid-19, diabetes, cough syrup etc.)	3%	1%	1%	4%
Supply of medicine (Malaria, Covid-19, etc.)	1%	0%	0%	1%
SRH education/services	1%	0%	0%	2%
Covid-19 testing	0%	1%	0%	0%

### Frequency of service

**Fig 3:**
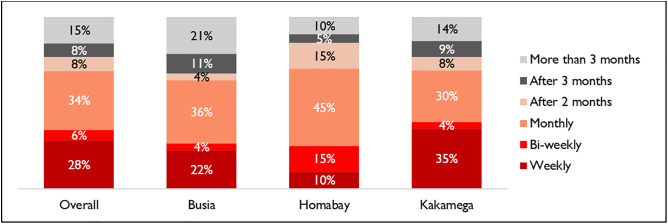
Frequency of services received from a CHV.

Fig 3 below captures responses on how frequently the respondents received services from CHVs. The most common frequency for service provision was monthly (38%), with the least common frequency being bi-weekly (6%). Comparison by county indicates that monthly CHV services were more common in Homabay (45%) than in Busia (36%) and Kakamega (30%). Weekly services were more common in Kakamega (35%) than in Busia (22%) and Homabay (10%).

**Fig 4:**
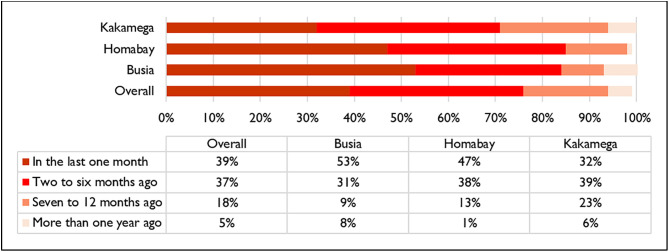
Timeline of the most recent malaria services received from a CHV.

Most respondents (39%) in this group had received a service from a CHV in the month leading up to the survey. As shown in Fig 4, a comparison across the counties revealed that in Kakamega, the proportion of those who had received a service in the last month (32%) was relatively less than that of Busia (53%) and Homabay (47%).

### Quality of service

**Fig 5:**
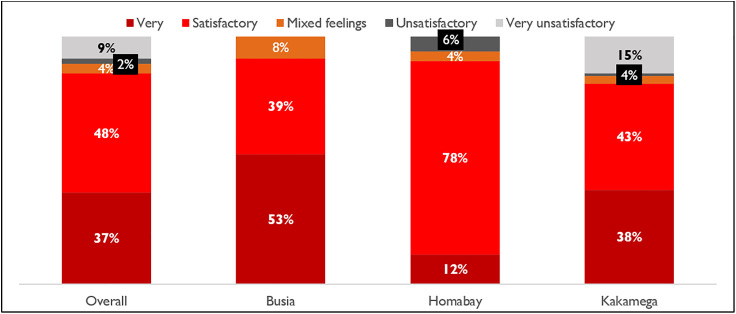
Levels of satisfaction with malaria services received from a CHV.

Overall, 85% of the respondents were either “satisfied” or “very satisfied” with the malaria services offered by CHVs. Fig 5 illustrates that respondents from Busia reported the highest overall satisfaction at 92% followed by Homabay at 90% and Kakamega at 81%.

To further measure satisfaction amongst those who had received malaria services from CHVs, respondents were asked to rate the service offered by CHVs on various aspects. When the high rating is based on the proportion of respondents who mentioned a service as being offered by CHVs every time, keeping information confidential by CHVs had the highest rating (63%), especially in Busia (72%) than Kakamega (69%) and Homabay (39%). Notably, Kakamega had the highest rating for almost all aspects that were rated except confidentiality, which stood out more in Busia; conversely, Homabay had the lowest rating in all rated aspects.

### Home treatment and its benefits

**Fig 6:**
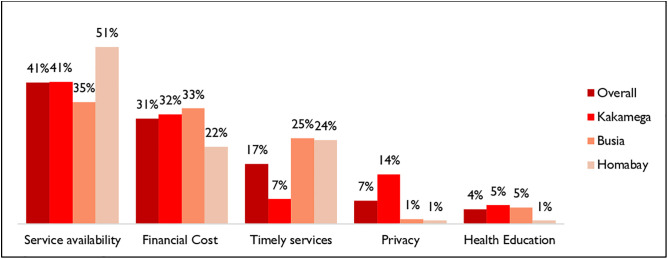
Most appreciated aspects of home treatment.

The survey sought to understand what respondents liked about home treatment, and verbatims were recorded. Fig 6 below gives a summary of themes grouped based on survey responses; after analyzing the findings captured by the chart, content analysis was done to shed light on what each encapsulates.

From a cursory look, overall, service availability was the most occurring theme at 41%, followed by financial cost at 31%, timely services at 17%, privacy at 7%, and health education at 4%. Comparison by county reveals that access to services was more pronounced in Homabay (51%) than in Kakamega (41%) and Busia (35%)

### Facilitators of effective CCMm

#### a) Service availability.

Home treatment services were mentioned to be fast, especially during an emergency, and were more convenient where the distance to the hospital was long.

*“Home treatment saves on the movement to hospital and back, sometimes the patient could be very sick and can’t sit on a motorcycle”* Respondent Busia

*“You get services at the comfort of your home”* Respondent Kakamega

CHVs were drivers to effective home treatment since they were able to reach large parts of the community, and they nurtured the confidence of the community regarding medical advice and follow-up after treatment.

*“They CHVs gives best services; they are the people we live together in the same community, so they usually follow up after treatment” Respondent Kakamega*

*“We readily get fast assistance in times of emergency. Some of us who rarely find time to visit hospitals readily find help from the CHVs”* Respondent Homabay

#### b) Financial cost.

Home treatment was mentioned as being economical to respondents since they would not incur transport costs, and medication was either cheap or free.

*“You don’t have to buy drugs, it’s cheaper compared to hospital”* Respondent Kakamega

*“The drugs and testing is free. They also register household for IRS and net distribution”* Respondent Homabay

#### c) Timely services.

While at home, services were quick, and respondents didn’t have to spend time queueing like in the hospital. In addition, home treatment services offered an opportunity to get faster instructions that did go through a layer of stages as the hospital.

*“We get fast health services, be it health education or even minor illnesses that she can handle. CHVs save us the time and money to access the hospitals”* Respondent Homabay

#### d) Privacy.

Respondents felt that whenever they interacted with a CHV, they could express themselves freely without fear of stigmatization, and CHVs were very confidential, with information being disclosed to them.

*“It’s comfortable especially for men because they fear going to hospital. It enhances privacy as it doesn’t alarm people in the village that you are sick because when you go to hospital everyone in the village will know”* Respondent Kakamega

*“….I like their confidentiality”* Respondent Busia

#### e) Health education.

While at home, community members were getting more information from CHVs; this can also be attributed to free interaction between CHVs and community members who had gained confidence in the privacy of their information. Further, frequent interaction with CHVs when following up on treatment further enhanced health education.

*“Regular checkups keep me well informed about my health status”* Respondent Homabay

*“Community members are educated on health by the CHV”* Respondent Kakamega

### What can be improved?

**Fig 7:**
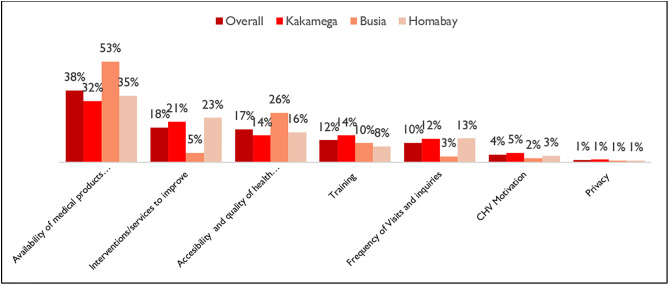
Suggested areas for improvement.

Fig 7 gives a summary on themes detailing what can be improved. Overall, the most occurring theme based on the proportion of the number of mentions was the availability of medical products and services (38%) followed by a theme detailing specific interventions/services to improve (18%), theme relating to accessibility and quality of services (17%), training (12%), frequency of CHVs visits (10%), need for CHV motivation (4%) and privacy (1%).

### Barriers to CCM

#### a) Availability of medical products and commodities.

Respondents were advocating for CHVs to be supplied with more drugs such as painkillers & antimalaria drugs; in addition, they want stability in the supply of drugs since the demand is high. Respondents also wanted CHVs to be equipped with tools such as thermometers, blood pressure monitors, weighing scales, and more mosquito nets.

*“CHVs to be given other medicines such as panadols, amoxicillin…”* Respondent Busia

*“Avail commodities to CHVs. drugs and testing kits”* Respondent Homabay

#### b) Interventions/services to improve.

Based on survey responses, intervention/services have been nested together. These include equipping and training CHVs to provide drugs for other illnesses such as blood pressure, free mosquito repellants, mosquito nets, outreach programs, and civic education on malaria treatment and prevention.

*“There is need for civic education about nets to educate people why they should not use them to cover chicken houses”* Respondent Kakamega

*“People need to be informed on spraying of mosquitoes regularly in addition distribution of enough mosquito nets”* Respondent Kakamega

The intervention also needs to be inclusive especially considering the elderly in the community during health education and teenage girls.

*“Health workers to give more health education to our teenage daughters”* Respondent Homabay

#### c) Accessibility and quality of health services.

Respondents advocated for increasing the number of CHVs and providing them with means of transport to respond quickly.

*“Provide means of transport to CHVS during monthly meetings”* Respondent Kakamega

The quality of medicine provided was also brought into the limelight following a recommendation to ensure that it is not expired and is effective.

#### d) Training.

Although, as previously mentioned, home-based treatment was received positively, some beneficiaries were not convinced of the knowledge of CHVs in giving medication and offering home-based treatment. Others indicated a need for CHVs to build confidence during home-based treatments. For example, a respondent described the need to train CHVs on handling pregnancy issues, extreme cases, and case management.

*“Equip CHVs with proper knowledge and information regarding community health matters”* Respondent Kakamega

*“Offer refresher courses to the CHVs”* Respondent Kakamega

*“CHVs should be trained to treat people at home”* Respondent Homabay

#### e) Frequency of visits.

CHVs were advised to offer regular visits to community members. Notably, they should avoid impromptu visits by ensuring that they first establish a person’s availability before visiting them. Observing time when visiting was also viewed as necessary.

*“The CHVs should announce the date for visiting them earlier so that they’ll be able to find them at home”* Respondent Kakamega

#### f) CHV Motivation.

Respondents advocated on providing (or increasing) monthly stipends to CHVs, also they should be paid on time without long delays.

*“Please take care of our CHVs in terms of late payments or failure to give them some little upkeep”* Respondent Homabay

## Discussion

The study sought to assess the effectiveness of CCMm and evaluate key facilitators and barriers across three high-burden counties in Western Kenya. Key findings are as discussed below:

### Knowledge, attitudes and practices

Improving knowledge, attitudes, beliefs, and practices emerged as pivotal for successful CCMm. All respondents were aware of Malaria with 97% of respondents citing mosquito bites as the main cause of malaria. This is consistent with previous studies that have reported mosquito bites as the main cause of Malaria [9,10]. Fever (89%), headache (71%), joint pains (61%), and vomiting (52%) were most frequently cited malaria symptoms. This affirms the broad awareness of malaria clinical symptoms among caregivers.

The study also found that the respondents were aware of the method used to prevent Malaria, the most cited method was sleeping under a mosquito net with women and children being given priority in the use of the mosquito nets. Past research has highlighted the importance of mosquito net use [11,12]. Draining of stagnant water as a preventive method was stated by only 43% of all respondents. Other preventive measures mentioned during KIIs and FGDs included Clearing nearby bushes, use of insecticides to kill mosquitos, vaccination against malaria for children under 2 years of age and use of prophylaxis malaria drugs during pregnancy. This suggests a need for further community sensitization on the causes of and prevention of malaria to dispel any misconceptions and improve knowledge.

### Satisfaction and uptake of services

The findings that majority of the respondents were satisfied with the malaria services offered by CHVs is consistent with other studies that have reported high levels of satisfaction with case management within the community [13–15]. Respondents from Busia reported the highest proportion of satisfaction compared to those in Kakamega and Homabay. This variation in satisfaction across the study sites can be attributed to differences in the availability and quality of service delivery across Counties.

The top three benefits of home treatment as perceived by the respondents were; service availability, financial costs, and timeliness of services. The respondents liked that the services were fast as opposed to the long procedures in health facilities. Home treatments were also economical since they didn’t have to incur transport costs and medication was cheap or free. This result suggests that improving service availability could enhance the uptake of community case management as access to health care services is a critical factor in improving health outcomes [16]. Reducing financial barriers can also enhance access to quality services [17].

### Facilitators of CCMm implementation

The survey sought to establish the factors that enable the implementation of CCMm. Some of the factors that supported implementation were the availability of commodities for malaria prevention and treatment, knowledgeable CHVs that have received adequate training, a functioning referral system where all cases of complicated malaria identified at the community can be referred to the health facility, the community’s trust in CHVs’ ability to treat them, and support supervision from the CHEWs and health management team. These enablers reflect broader findings from other scholars who emphasized commodity availability and trust in frontline health workers as foundational to effective CCMm delivery [18,19].

### Barriers to CCMm implementation

Despite these strengths, the study also identified critical barriers. For instance; not all CHVs had received training on CCMm, non-adherence to mosquito net use due to myths and misconceptions surrounding the use of mosquito nets, stock out of malaria commodities disrupted service continuity, and in some areas some facilities are not up to standard to support CCMm. Inaccessible roads, healthcare worker strikes and absence of reporting tools used to refer patients further hindered the patient referral system. These findings are consistent with past research [19]. Data inaccuracy and incomplete reporting by CHVs also emerged as a concern, limiting the reliability of service tracking.

Another critical barrier was the lack of regular stipends or incentives for the CHVs which can negatively impact morale and job satisfaction. This can lead to a shortage of trained CHVs and a reduction in the quality of care provided. This is consistent with findings [20] that incentives are essential for sustaining CHV motivation and performance.

Comparison by county revealed that access to services was more pronounced in Homabay than in Kakamega and Busia. These findings highlight the importance of contextual factors such as geographical location in understanding the facilitators and barriers of CCMm.

This study had some limitations. Being a cross-sectional study limits causal inferences. Data also relied heavily on self-reporting by caregivers which may introduce recall bias. Only one caregiver per household was interviewed, potentially overlooking other perspectives. Future research should explore longitudinal design to track CCMm outcomes over time and assess sustainability.

## Conclusion

In conclusion, this study has identified several barriers and enablers in the uptake of CCMm. Improving knowledge, attitudes, beliefs, and practices is pivotal for the successful implementation of CCMm. While the study found that there is a good understanding of the causes and symptoms of malaria, there is need for further community sensitization on the causes and prevention of malaria to dispel any misconceptions and improve knowledge. Satisfaction with the services offered by CHVs was high with service availability being the top benefit of home treatment.

Factors supporting the implementation of CCMm included adequate training for CHVs, commodity availability, and a functioning referral system. However, the study also highlighted barriers such as stock-outs of malaria commodities, inadequate facilities, Job dissatisfaction among CHVs, and HCW strikes that hindered the implementation of CCMm. These factors undermine the continuity and quality of care.

To strengthen CCMm it is essential that stakeholders involved in malaria control programs invest in sustainable support mechanisms, ensuring reliable commodity supply, fair compensation for CHVs, improved infrastructure and enhanced community health education. Addressing these gaps is critical for advancing equitable community-based malaria management and improving health outcomes in endemic regions.

## Supporting information

S1 File Study Dataset (Excel format). (XLSX)

S2 File Data Collection Tools (ZIP file containing questionnaires and tools). (ZIP)
