# Supplementary material for: Facilitators and barriers of Community Case management of Malaria implementation in Homabay, Busia and Kakamega Counties, Kenya
Source: PLoS One. 2025 Aug 21;20(8):e0329709. doi: 10.1371/journal.pone.0329709 (PMC12370068; doi:10.1371/journal.pone.0329709)
Supplement: S2 File — (ZIP) [file pone.0329709.s002.zip › Malaria Household Survey Questionairre.docx]

Household Survey Tool

Section 1: Demographics (Respond to all Questions)

| 1.1 | Consent notes of respondent | Agree 2. Not Agree | | | | | | | | | |
| --- | --- | --- | --- | --- | --- | --- | --- | --- | --- | --- | --- |
| 1.2 | Date of Interview |  | | | | | | | | | |
| 1.3 | Name of Respondent |  | | | | | | | | | |
| 1.4 | Age of Respondent (Actual) |  | | | | | | | | | |
| 1.5 | Sex of respondent (Observe) | Male  Female | | | | | | | | | |
| 1.6 | Respondent level of education | Never gone to school  Primary  Secondary  College  University  Postgraduate | | | | | | | | | |
| 1.7 | Respondent marital status | Married  Single  Divorced/ separated  Widowed/ Widower | | | | | | | | | |
| 1.8 | County | Bungoma  Busia  Homabay  Kakamega  Migori  Siaya | | | | | | | | | |
| 1.9 | Subcounty |  | | | | | | | | | |
| 1.10 | How many people live in  this Household (including  children and Head of  Household |  | | | | | | | | | |
| 1.10 | Household Size: No. of people living in HH by age | < 5 yrs | | 6 – 17 Yrs | | 18 – 35 yrs | | 36 – 60 yrs | | > 60 yrs | |
|  |  | M | F | M | F | M | F | M | F | M | F |
|  |  |  |  |  |  |  |  |  |  |  |  |

Section II: Malaria Knowledge, Attitude, Beliefs and Practice

| 2.1 | 0.Have you ever heard of an  infection called malaria | Yes  No |
| --- | --- | --- |
| 2.2 | What causes Malaria | Mosquito bites………………  Drinking dirty water………...  Staying in the cold…………..  Being rained on……………..  DON’T KNOW ………………  Other |
| 2.3 | 2.What are the common  signs/symptoms of malaria? | Fever ………………………….  Joint pains…………………….  Headache………………………  Poor appetite…………………..  Vomiting………………………  Diarrhoea……………………….  DON’T KNOW |
| 2.4 | Is it possible for a healthy-looking person to have malaria | Yes  No |
| 2.5 | What are the main ways of preventing malaria? (Multiples options allowed) | Sleeping under a mosquito  net…  Draining stagnant water around  the  compound………………….  Using mosquito repellents…….  DON’T KNOW |
| 2.6 | Has any of the people you stay within your household been ill  with fever at any time in the last 6 months | Yes  No |
| 2.7 | Did you seek advice or  treatment for the illness from any source | Yes  No |
| 2.8 | Where did you seek advice or treatment? | Shop………………………..  Government hospital/Health  Center/Dispensary…………..  Private hospital/clinic……….  Private pharmacy……………  Friend/Relative……………...  Traditional herbalist…………  CHV…………………………  Other(Specify) |
| 2.9 | Where did you first seek advice  or treatment? | Shop………………………..  Government hospital/Health  Center/Dispensary…………..  Private hospital/clinic……….  Private pharmacy……………  Friend/Relative……………...  Traditional herbalist…………  CHV…………………………  Other  (specify… |

Section III: Community Case Management of Malaria

| 3.1 | Have you heard about Community Health Volunteers | Yes  No |
| --- | --- | --- |
| 3.2 | Have you or any of your  household member ever received  health services from a CHV? | Yes  NO |
| 3.3 | Which services did you or your household member receive from the CHV? | WASH…………………  HIV/AIDS………………  TB………………………  MNCH…………  Nutrition…………………  Malaria…………………  Health education………………...  Other (specify… |
| 3.4 | How often do you get services from a CHV? | Monthly…………………  After 2 months………………  After 3 months……………  More than 3 months………… |
| 3.5 | Overall, how do you feel about the services you or your household member received from a CHV? Would you say it is very satisfactory, satisfactory, mixed feelings, unsatisfactory, very unsatisfactory | Very  Satisfactory………………  Satisfactory……………  Mixed feelings…………………  Unsatisfactory………  Very unsatisfactory…… |
| 3.6 | Do you consider a CHV as your regular source of health care? | Yes  No |
| 3.7 | Have you or any of your  household member ever received malaria services from a CHV? | Yes  No |
| 3.8 | Which malaria services did you receive? | Testing…………………  Treatment using ACT or AL…….  Referral…………………  Net distribution………………  Health education |
| 3.9 | When did you or your household member last receive malaria services from a CHV? | In the last one month……………  Two to six months ago………….  Seven to 12 months ago…………  More than one year ago……… |
| 3.10 | Overall, how do you feel about  the malaria services you or your  household member received from  a CHV? Would you say it was very satisfactory, satisfactory, mixed feelings, unsatisfactory, very unsatisfactory? | Very  Satisfactory………………  Satisfactory………………  Mixed feelings…………………  Unsatisfactory……………  Very unsatisfactory… |
| 3.11 | Please cycle what you feel/think about the following on the malaria services offered by CHVs: | |
| 3.11 a | Is available when needed | Always…………………  Usually,…………………  Sometimes………………  Never…………………… |
| 3.11 b | Promptly returns calls (quick to act, timeliness, ready in action) | Always…………………  Usually,…………………  Sometimes………………  Never…………………… |
| 3.11 c | Listens to you | Always…………………  Usually,…………………  Sometimes………………  Never…………………… |
| 3.11 d | Takes enough time with  you | Always…………………  Usually,………………  Sometimes………………  Never…………………… |
| 3.11 e | Provides you with  information on  procedures, diagnosis,  treatment | Always…………………  Usually,………………  Sometimes………………  Never…………………… |
| 3.12 d | Answers your questions | Always…………………  Usually,………………  Sometimes………………  Never…………………… |
|  | Keeps your information  in confidence | Always…………………  Usually,…………  Sometimes………………  Never…………………… |
|  | Is helpful to you | Always…………………  Usually,…………  Sometimes………………  Never…………………… |
|  | Follows up to ensure  healing | Always…………………  Usually,……………  Sometimes………………  Never…………………… |
| 3.12 | What do you like best about the home treatment? |  |
| 3.13 | What do you like least about home treatment? |  |
| 3.14 | Suggestions for improvement or any other comment? |  |
