# Supplementary material for: Facilitators and barriers of Community Case management of Malaria implementation in Homabay, Busia and Kakamega Counties, Kenya
Source: PLoS One. 2025 Aug 21;20(8):e0329709. doi: 10.1371/journal.pone.0329709 (PMC12370068; doi:10.1371/journal.pone.0329709)
Supplement: S2 File — (ZIP) [file pone.0329709.s002.zip › Community members FGD guide.docx]

Community Members FGD Guide

1. What malaria preventive measures are in place at the community level?
2. What are the existing barriers and facilitators to CCMm? Explore Community factors, Health Infrastructure, CHVs
3. Are there any gender dynamics that impact on use of mosquito nets at the household level? Explore Culture, Knowledge, Attitude and Practice, Accessibility, Availability, affordability, Inequality?
4. What would you consider as the contribution of community case management of malaria (CCMM) in prevention and control of Malaria in this community?
5. What would you consider as practices that need to be maintained in the implementation of the project?
6. Has the project been able to address the health needs of the populations served? Probe: what need to be improved to enable the target populations realize the benefits of the project?
7. Are there noticeable changes brought about by the project among the community served?
8. Do you think the project’s approach would be relevant elsewhere? Yes/No. Why/Why not
