# Supplementary material for: Facilitators and barriers of Community Case management of Malaria implementation in Homabay, Busia and Kakamega Counties, Kenya
Source: PLoS One. 2025 Aug 21;20(8):e0329709. doi: 10.1371/journal.pone.0329709 (PMC12370068; doi:10.1371/journal.pone.0329709)
Supplement: S2 File — (ZIP) [file pone.0329709.s002.zip › Health workers KII Guide.docx]

KII Guide-Health workers-Malaria

1. How is the Community uptake of CCMm?
2. What malaria Preventive measures are in place at the community level? Explore Malaria vaccine, iPTP, commodities and CHV toolkits
3. What is the effectiveness of existing malaria management initiatives? (Probe on: Knowledge, drugs, facility, CHVs)
4. What are the existing barriers and facilitators to CCMm? (Probe on: Community factors, Health Infrastructure, CHVs
5. Are there any gender dynamics that impact on use of mosquito nets at the household level? (Probe on: Culture, Knowledge, Attitude and Practice, Accessibility, Availability, affordability, Inequality)
6. Who is most at risk and what strategies work best for each category?
7. Is community data on CCMm available and how is it used for decision making?
8. What are the challenges faced in data management in the CCMm program?
